# Long-term effects of nurse-led individualized education on middle-aged patients with acute coronary synrome: a quasi-experimental study

**DOI:** 10.1186/s12912-017-0254-y

**Published:** 2017-10-16

**Authors:** Jae Lan Shim, Seon Young Hwang

**Affiliations:** 1 Department of Nursing, Doowon Technical University, 51, kwaneumdang-gil, Juksan-myon Anseong-si, Gyeonggi-do, 17520 South Korea; 2 College of Nursing, Hanyang University, 222 Wangsimniro, Seondong-gu, Seoul 04763 South Korea

**Keywords:** Acute coronary syndrome, Self-care compliance, Self-efficacy, Patient education, Quality of life

## Abstract

**Background:**

This study examined the long-term effects of nurse-led, individualized education on self-efficacy, self-care compliance, and health-related quality of life (HRQoL) in middle-aged patients with new-onset acute coronary syndrome**.**

**Methods:**

A quasi-experimental pretest-posttest design was used in the study. A cardiovascular nurse provided individualized education to the intervention group (*n* = 32), and self-efficacy, self-care compliance, and HRQoL at baseline and 3 and 12 months after discharge were compared to those of a control group (*n* = 30). Patients were recruited from a cardiovascular care unit at a university hospital between 2012 and 2013. Repeated measures analysis of variance was used to compare time-related changes.

**Results:**

There was no significant difference in self-efficacy between the 2 groups over a fixed period (*F* = 3.47, *p* = 0.067) and showed no interaction between the 2 groups (*F* = 0.45, *p* = 0.636). However, significant differences were found in the main and interaction effects between the 2 groups and changes in self-care compliance over the follow-up period differed significantly between the 2 groups (*F* = 28.72, *p* < 0.001). Changes in mental HRQoL over the follow-up period differed significantly between the 2 groups (*F* = 33.34, *p* < 0.001) and significant interaction effect (*F* = 4.40, *p* = 0.040).

**Conclusions:**

Structured nurse-led education should be provided to middle-aged patients with new-onset acute coronary syndrome, as part of routine predischarge education, to increase self-care compliance and mental HRQoL and prevent secondary adverse events.

**Trial Registration:**

Trial registration number (TRN) is KCT0002454. The study was registered retrospectively with registration date July 9, 2017.

## Background

Acute myocardial infarction (AMI) and unstable angina exist on a spectrum of clinical diseases collectively identified as acute coronary syndrome (ACS). The mortality rate for patients with ACS has decreased because of thrombolytic agents and advances in treatments such as surgical reperfusion [1]. In addition, the long-term prognosis has been shown to be excellent in hospital survivors, and the 1-year survival rate is 89.5% [2]. However, 18.4% of patients are at risk of subsequent major adverse cardiac events.

According to the Statistics Office of Korea [3], heart disease was the second leading cause of death in the country in 2015, with 55.6 deaths per 100,000 population, and ischemic heart disease accounted for 55.2% of cardiovascular deaths. The 1-year mortality rate for patients with AMI who had received acute treatment was shown to be 8.3%, and patients with recurrent AMI exhibited an increased risk of death relative to that of those with new-onset AMI [4].

To prevent the occurrence of major adverse cardiac events (MACE), particularly re-stenosis, subsequent interventions should focus on behavioral modification and the reduction of risk factors for cardiovascular disease. Interventions should also strive to secure optimum health-related quality of life (HRQoL) by helping patients to recover their physical, social, and mental function following ACS [5]. Previous studies have shown that patients who adhered to self-care regimens during the acute and recovery stages of ACS reduced their risk of CVD by modifying unhealthy habits and behavior [6]. A previous study involving patients with ACS showed that self-efficacy exerted a direct effect on self-care compliance [7]. Therefore, education programs that focus on the prevention of a second acute coronary event and improvement of lifestyle habits should emphasize the improvement of self-efficacy to comply with self-care regimens and promote healthy behavior.

In Korea, adult patients with ACS aged 65 years or younger tend to exhibit a significantly higher number of lifestyle-related cardiovascular risk factors, including smoking, binge drinking, stress, unhealthy diet, and hyperlipidemia, relative to those observed in older patients [8]. In addition, middle-aged individuals in the most productive age bracket are focused on their careers and become vulnerable to stress and chronic health problems. Nonetheless, the vast majority of people in this age group have been shown to exhibit poor awareness of the seriousness of CVD [8]. A meta-analysis of 18 studies showed that the presence of even a single risk factor in individuals in their 40s or 50s dramatically increased their lifetime risk of CVD, relative to that observed in individuals with no risk factors [9]. According to the Korea Acute Myocardial Infarction Registry Study, 62.4% of 39,978 patients with ACS who were treated between 2005 and 2013 were men, and 46% were current smokers. Smoking has also been shown to be the most frequent cause of AMI in people younger than 65 years of age [10]. Moreover, another study reported that smokers with AMI who received percutaneous coronary intervention (PCI) were younger relative to nonsmokers, whose mean age was 58 years, and the 1-year mortality rate for the smoking group was significantly higher relative to that observed for the nonsmoking group [11]. Therefore, the importance of reducing lifestyle risk factors to prevent secondary attacks should be emphasized for middle-aged patients with new-onset ACS.

As mentioned, ACS is an acute disease that requires consistent education and management to prevent recurrent events and complications. Therefore, patient education should focus on an active patient role in disease management via self-care compliance. We believe that nurses play a key role in providing education regarding certain issues, such as risk factors and signs of MACE, for patients and their caregivers. Furthermore, nurses are well placed to communicate the need to maintain self-care to the clinical team. Practical and effective nursing interventions should be tailored to individuals’ educational needs and aim to reduce cardiovascular risk factors [12]. Yıldız et al. [13] found that strong individualized education provided prior to discharge following cardiac intervention improve quality of life (QoL) and relieved anxiety related to postdischarge self-care practices. Therefore, nurse-led, individualized education could be the most effective strategy for ACS patients with respect to promoting lifestyle modification.

To examine the above-mentioned issues, we provided middle-aged patients with new-onset ACS with nurse-led, individualized education prior to discharge, with the aim of preventing a secondary attack. Specifically, we sought to test the following hypotheses:

- **Hypothesis 1:** Nurse-led, individualized education will affect self-efficacy in patients with ACS for up to 1 year subsequent to discharge.
- **Hypothesis 2:** Nurse-led, individualized education will affect self-care compliance in patients with ACS for up to 1 year subsequent to discharge
- **Hypothesis 3:** Nurse-led, individualized education will affect HRQoL in patients with ACS for up to 1 year subsequent to discharge.

## Methods

### Study design

We explored the effects of individualized education for up to 1 year, in patients with ACS who were due to be discharged and received PCI concerning self-efficacy, self-care, and HRQoL. The study used a nonequivalent, control group, pretest-posttest design.

### Study participants

**Fig. 1:**
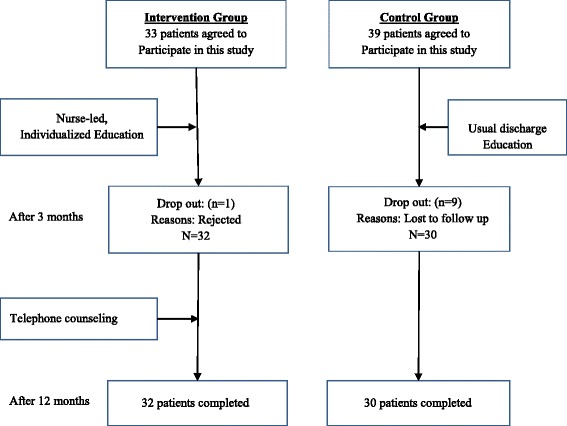
Flow chart of the study design and data collection

The participants were middle-aged patients with new-onset ACS who were initially hospitalized at a university hospital in Korea between May and November in 2012 and underwent 1-year follow-up assessment in 2013. Patients who had been hospitalized between May and July were assigned to the control group, and those who were hospitalized between September and November were assigned to the intervention group. The inclusion criteria were as follows: age of 40–65 years, imminent discharge following a first PCI, ability to communicate effectively and complete a self-report questionnaire accurately, ability to understand the purpose of the study, and consent to participation. Sample size calculation showed that the minimum of participants required in each group was in a repeated measures analysis of variance with power (1-β) of .80, a medium effect size of .25, three measurements, and a significance level of α = .05, was 19 based on a previous study [14]. Therefore, we recruited 32 patients for the intervention group and 30 patients for the control group, which was sufficient to achieve statistical power. Figure 1 shows the flowchart for the study.

### Intervention: Nurse-led, individualized education

A cardiovascular nurse provided participants with individualized education and counseling. The intervention group participated in counseling and interviews for approximately 40 min, and their family members were encouraged to participate. The program was divided into 2 phases. In the first phase, we focused on identifying patients’ needs and characteristics and provided an overview of ACS risk factors. In the second phase, we discussed exercise, diet, medication, stress management, smoking management, and health behavior strategies, with the aim of gaining the support of participants and their family members. Upon discharge, we provided patients in both groups with an educational multimedia compact disc containing information designed to encourage them to manage ACS-related risk factors at home. In addition, 2 telephone-based follow-up counseling sessions were provided by the nurse between 5 and 10 months subsequent to discharge.

### Measurement

#### Self-efficacy

Self-efficacy was measured using an assessment tool developed by Song [15] and based on the American Heart Association’s Cardiovascular Risk Factor Assessment Tool [16]. The scale consists of 19 items pertaining to medication compliance (5 items), diet (4 items), exercise (4 items), and smoking cessation (5 items). Responses are provided using a 4-point Likert scale ranging from 1 (never) to 4 (very often). Total scores range from 19 to 76, and higher scores indicate greater self-efficacy. Cronbach’s α for the scale was .91.

#### Self-care compliance

Self-care compliance was measured using an assessment tool developed by Park [17] and modified by Son [18]. The scale consists of 23 items pertaining to medical checkups and medication (5 items); dietary habits (7 items); alcohol consumption and smoking (2 items); exercise and rest (4 items); sexual intercourse (1 item); stress management (1 item); and checking blood pressure (1 item), pulse (1 item), and weight (1 item). Responses are provided using a 5-point Likert scale, and higher scores indicate greater compliance with self-care. Cronbach’s α for the scale was .87.

#### HRQoL

HRQoL was measured using the Short Form-36, version 2 (SF-36, v2), which was developed by Ware and Sherboune [19]. The Optum company granted permission for the use of the tool. The scale includes 36 items divided between the following 8 subscales: physical function (10 items), physical limitation (4 items), physical pain (2 items), general health (5 items), vitality (4 items), social function (2 items), emotional limitations (3 items), mental health (5 items), and health change (1 item). The item scores are divided into a physical component summary (PCS) score and a mental component summary (MCS) score. Higher scores for all subscales indicate higher levels of HRQoL, with the exception of the physical pain and physical limitation subscales, for which the opposite is true. Cronbach’s α for the scale was .81 in the current study, and the scale demonstrated reliability and validity in a study conducted by Koh et al. [20].

#### General and ACS-related characteristics

With respect to participants’ general characteristics, we measured age, sex, educational level, economic status, marital status, family history, employment status, and religion. Regarding disease-related characteristics, we used medical records to identify ACS risk factors including smoking status, the presence or absence of pulmonary edema, and total cholesterol levels.

### Data collection procedure

Initial data for the pretest were collected between May and December 2012. Participants were provided with questionnaires upon visiting the outpatient department and 3 and 12 months subsequent to the individualized education, via 2 follow-up calls made by cardiovascular nurses. The control group was also provided with a self-study CD and instructions for usual care following discharge. Initially, the intervention and control groups each included 40 patients; however, only 33 and 39 patients in the intervention and control groups, respectively, provided written consent for study participation. At the 3-month follow-up assessment, 1 patient from the intervention group refused to participate in the study, and 9 patients in the control group were uncontactable. Ultimately, 32 and 30 patients in the intervention and control groups, respectively, participated in the study.

### Data analysis

Data were analyzed using SPSS WIN 21.0. Participants’ general characteristics, disease state, physiological index, and self-efficacy and the homogeneity of self-care compliance were assessed using t tests or chi-square tests. Paired t tests were used to compare changes in self-efficacy, self-care compliance, and HRQoL from baseline to 3 and 12 months subsequent to the nurse-led, individualized education. A repeated measures analysis of variance was performed to examine the effectiveness of individualized education in the intervention and control groups during the follow-up period.

## Results

### Homogeneity

Participants’ characteristics are shown in Table 1. Patients’ mean ages in the intervention and control groups were 51.92 ± 6.13 and 50.42 ± 6.02 years, respectively. In addition, 92.3% and 87.9% of participants in the intervention and control groups, respectively, were men. With respect to disease-related characteristics, 53.8% of the intervention group and 57.6% of the control group were patients with ST segment elevation myocardial infarctions, and 28.3% of the intervention group and 27.2% of the control group were non-ST segment elevation myocardial infarction patients. Regarding smoking, 46.2% of the intervention group and 51.5% of the control group were current smokers. General and ACS-related characteristics did not differ significantly between the 2 groups, indicating that the 2 groups were homogeneous (Table 1). In addition, self-efficacy, self-care compliance, and HRQoL did not differ significantly between the 2 groups, indicating homogeneity (Table 2).

**Table 1:** Homogeneity of baseline characteristics between the groups

Variables	Categories	Intervention	Control	t or χ^2^	*P*
		*n* = 32(%)	*n* = 30(%)		
Age (yr)	M ± SD	51.92 ± 6.13	50.40 ± 6.02	−0.97	0.334
Gender	Male	30 (92.3)	26 (87.9)	0.09	0.929
Education level	≤High school	17 (51.3)	22 (72.7)	3.45	0.063
	≥ College	15 (48.7)	8 (27.3)		
Monthly income (USD)	≤ 3000	14 (43.7)	16 (54.5)	0.86	0.354
	≥3000	18 (56.3)	14 (45.5)		
Marital status	Married	30 (92.3)	26 (87.9)	4.08	0.253
	Unmarried/ Divorced	2 (7.7)	4 (12.1)		
Employed	Yes	30 (92.3)	24 (78.8)	1.25	0.159
Religious	Yes	15 (48.7)	16 (54.5)	0.24	0.622
Final diagnosis	UAP	6 (17.9)	5 (15.2)	0.41	0.813
	STEMI	17 (53.8)	17 (57.6)		
	NSTEMI	9 (28.3)	8 (27.3)		
Killip Class	I	25 (76.9)	23 (75.8)	1.89	0.579
	II-IV	7 (23.1)	7 (24.2)		
CVD family history	Yes	14 (43.7)	9 (30.3)	1.35	0.246
Total length of hospital stay	M ± SD	6.23 ± 2.92	6.43 ± 3.39	0.26	0.799
Smoking status	Never	10 (31.3)	8 (27.3)	0.26	0.878
	Quit	8 (25.0)	6 (21.2)		
	Current	14 (43.7)	16 (51.5)		
Total cholesterol	M ± SD	200.73 ± 46.61	194.72 ± 35.54	−0.56	0.142
Triglycerides	M ± SD	173.34 ± 114.33	167.29 ± 88.62	−0.23	0.577

UAP = Unstable angina pectoris, STEMI = ST segment elevation myocardial infarction, NSTEMI = Non-ST segment elevation myocardial infarction, SD = Standard deviation

**Table 2:** Homogeneity of dependent variables between the groups

Variables	Intervention (*n* = 32)	Control (*n* = 30)	t	*p*
	M ± SD	M ± SD		
Self-efficacy	56.51 ± 10.60	54.35 ± 10.58	- 0.79	0.946
Self-care compliance	68.85 ± 13.61	64.69 ± 10.53	- 1.30	0.576
Health-related QoL				
Physical health	50.24 ± 6.43	50.45 ± 7.12	- 0.69	0.891
Mental health	47.22 ± 9.14	44.85 ± 9.72	- 1.36	0.190

SD = Standard deviation, QoL = Quality of life

### Hypothesis testing

We rejected Hypothesis 1, in which self-efficacy scores in the intervention group were expected to differ from those of the control group over time. There were no significant differences in the main effect between the 2 groups over a fixed period (*F* = 3.47, *p* = 0.067) and showed no interaction between the 2 groups (*F* = 0.45, *p* = 0.636). However, self-efficacy scores differed significantly between the 2 groups when the groups were homogeneous (*F* = 15.56, *p* < 0.001), and there was no difference in changes in self-efficacy over the follow-up period between the 2 groups.

Hypothesis 2, in which self-care compliance scores for the intervention group were expected to differ from those of the control group over the follow-up period, was supported. Self-care compliance scores differed significantly between the control and intervention groups when the 2 groups were homogeneous (*F* = 7.08, *p* = 0.010). In addition, there was a significant interaction effect between the 2 groups, and changes in self-care compliance over the follow-up period differed significantly between the 2 groups (*F* = 3.59, *p* = 0.050). There were significant differences in the main effect between the 2 groups when time was fixed (*F* = 28.72, *p* < 0.001).

**Fig. 2:**
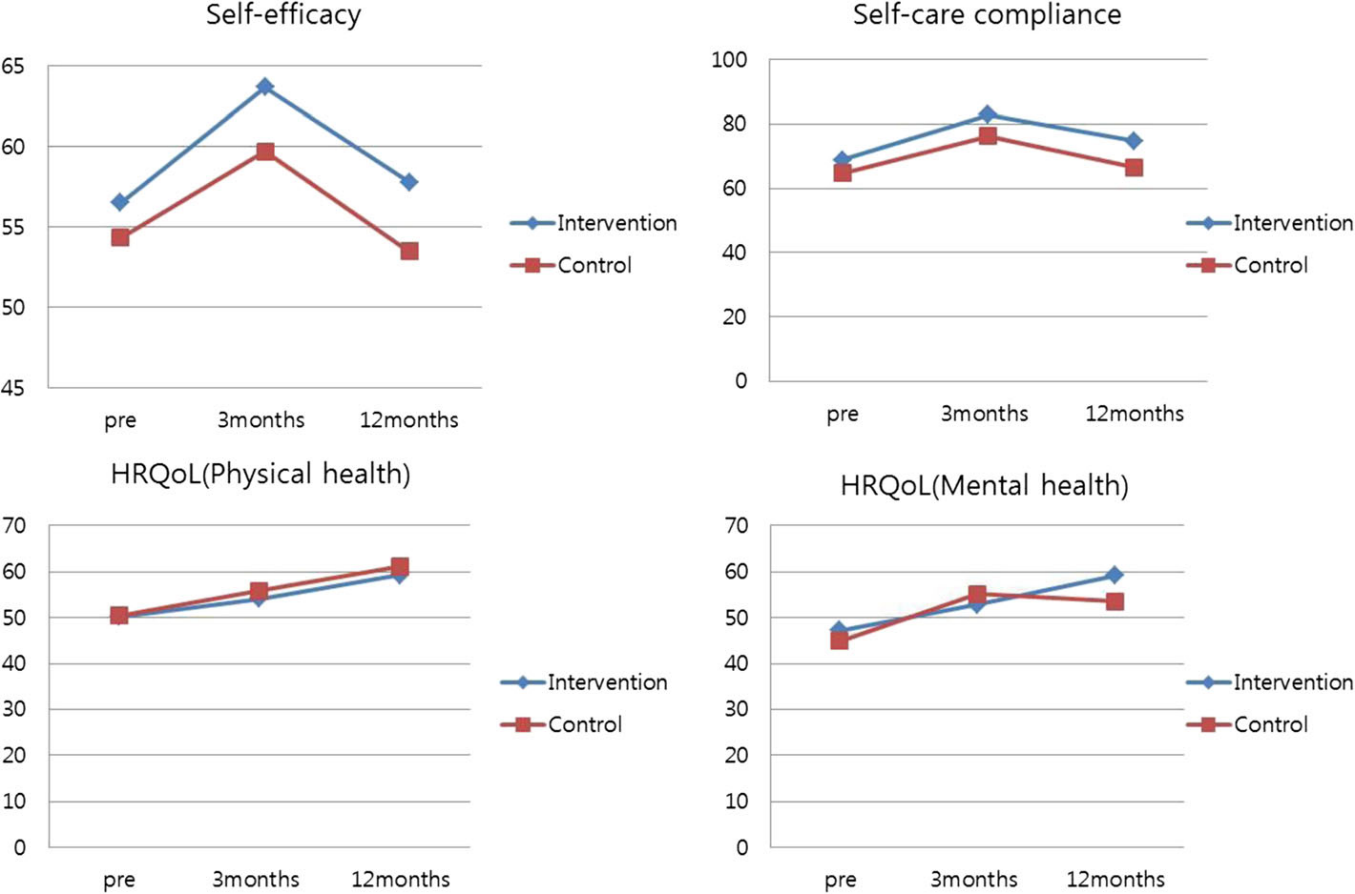
Changes in self-efficacy, self-care**,** physical and mental health-related QoL between the two groups across time

Hypothesis 3, in which HRQoL scores in the intervention group were expected to differ significantly from those in the control group over the follow-up period, was partially supported. PCS scores differed significantly between the control and intervention groups over time when the 2 groups were homogeneous (*F* = 18.27, *p* < 0.001). However, there were no significant differences in the main effect between the 2 groups when time was fixed (*F* = 0.35, *p* = 0.583). MCS scores differed significantly between the 2 groups over the follow-up period when the 2 groups were homogeneous (*F* = 33.34, *p* < 0.001), and there was a significant interaction between time and the 2 groups (*F* = 4.40, *p* = 0.040). However, there were no significant differences in the main effect between the 2 groups when time was fixed (*F* = 0.14, *p* = 0.713). The results of hypothesis testing have shown in Table 3 and Fig. 2.

**Table 3:** Effect of nurse-led individualized education on self-efficacy, self-care compliance, and health-related quality of Life

Variables	Group	Pretest	Posttest 1 (after 3 mo)	Posttest 2 (after 12 mo)	Source	F	*p*
		M ± SD	M ± SD	M ± SD			
Self-efficacy	I (*n* = 32)	56.51 ± 10.62	63.70 ± 4.82	57.83 ± 10.74	Group	3.47	0.067
	C (*n* = 30)	54.35 ± 10.61	59.73 ± 5.44	53.51 ± 10.43	Time	15.56	<0.001
					Group × Time	0.45	0.636
Self-care compliance	I (*n* = 32)	68.85 ± 13.63	82.82 ± 5.83	74.83 ± 16.73	Group	7.08	0.010
	C (*n* = 30)	64.69 ± 10.53	76.33 ± 5.16	66.54 ± 10.61	Time	28.72	<0.001
					Group × Time	3.59	0.050
HRQoL							
Physical health	I (*n* = 32)	50.24 ± 6.43	54.18 ± 6.02	59.28 ± 6.14	Group	0.35	0.583
	C (*n* = 30)	50.45 ± 7.12	55.88 ± 2.77	61.21 ± 3.05	Time	18.27	<0.001
					Group × Time	1.14	0.289
Mental health	I (*n* = 32)	47.22 ± 9.14	52.80 ± 8.51	59.2 ± 8.47	Group	0.14	0.713
	C (*n* = 30)	44.85 ± 9.72	55.17 ± 5.21	53.5 ± 9.46	Time	33.34	<0.001
					Group × Time	4.40	0.040

SD = Standard deviation, I: Intervention group, C: Control group, HRQoL = health-related quality of life

## Discussion

In the current study, nurse-led, individualized education was provided for middle-aged patients who had experienced new-onset ACS, prior to discharge. Two follow-up calls were made during the 1-year period following discharge. The results showed that self-efficacy did not differ significantly between the 2 groups during the follow-up period. This finding supports those reported by Taft et al. [21], which showed that cardiac self-efficacy following individualized education did not differ significantly between control and intervention groups. However, some previous studies have reported different results. For example, in one study, self-efficacy exerted the strongest effect on self-care compliance in patients with acute myocardial infarction [22]. These contradictory findings highlight the necessity for future research. In the current study, self-efficacy scores in the intervention and control groups had increased during the first 3 months following discharge and decreased 12 months subsequent to discharge. Although patients reported that they had strong purpose and the will to live because they had acquired information via individualized education following acute treatment, they became less motivated to maintain their health after experiencing little improvement.

The intervention group showed significantly greater differences in self-care compliance at 3- and 12-month follow up relative to that observed in the control group. This finding supports those of a previous study conducted by Uysal and Ozcan [23], which involved a group of AMI patients, of whom 75% and 71% were men and younger than 65 years of age, respectively. In the study, the patients were provided with approximately 1 h of individualized education via a brochure that provided information regarding disease, stress management, lifestyle modification, and diabetes control. As a result, the increase in physical activity and decrease in waist circumference in the intervention group were greater relative to those observed in the control group. Moreover, the findings of the current study are consistent with those of Mok et al.’s study [24], which showed that AMI patients who had received nurse-led, individualized education and counseling and telephone follow up adopted healthier diet habits, such as low-fat and -salt diets, and exhibited greater improvements in high-density lipoprotein cholesterol levels 3 months later. In addition, a systematic review and meta-analysis conducted by Fredericks and Yau [25] showed that individualized education regarding coronary artery disease was effective at improving compliance with a self-care program consisting of a diet regimen, medicine, an exercise regimen, risk factor modification, self-assessment, and reductions in hospital readmissions. Therefore, the results of the current study emphasize the ongoing need for the provision of nurse-led, individualized education prior to discharge, to prevent a secondary attack through lifestyle modification, particularly for middle-aged patients with ACS.

To determine the effects of individualized education on HRQoL, we obtained PCS and MCS scores. The results showed that PCS scores tended to increase with time in both groups and did not differ significantly between the 2 groups. A previous study conducted by Wong and Chair [26] showed that QoL tended to improve until 1 month subsequent to the PCI. It also showed that PCS and MCS scores began to decrease 3 months subsequent to the PCI, which emphasized the need for supportive, long-term nursing interventions to maintain improvements in QoL. In the current study, mental HRQoL (represented by MCS scores) increased in both groups until 3 months subsequent to the intervention, following which a gradual decrease in MCS scores was noted in control group. However, the intervention group showed a consistent increase in scores until 12 months later, indicating significant differences between the 2 groups and in the intervention group over time. These findings could be attributed to the long-term telephone counseling and individualized education provided in the study. However, in a study involving 288 patients with AMI who received individualized education via telephone 3 and 6 months subsequent to discharge and completed the SF-36, physical HRQoL differed significantly between the control and intervention groups. However, mental HRQoL did not differ significantly between the 2 groups [27]. In the current study, nurse-led, individualized education did not impact on significant difference between the 2 groups. One possible reason for this finding is that physical health was maintained because of the natural physical recovery process over time.

The main limitation of the study was that the participants were recruited from a single academic medical center; therefore, the findings were not generalizable to all patients with ACS in Korea. Furthermore, data were collected using self-report questionnaires and did not consider physiological parameters or clinical outcomes. Future collaborative studies examining this population should include changes in physiological indicators and MACE, such as readmission or mortality, over a longer period.

## Conclusions

The current study provided a basis for useful nursing strategies via which nurse-led, individualized education is provided for middle-aged patients with new-onset ACS, prior to discharge. Counseling provided via follow-up calls also appeared to improve the mental HRQoL by enhancing self-care compliance. Therefore, structured, individualized education should be provided to middle-aged patients with ACS by cardiovascular nurses, as part of routine predischarge education, to prevent secondary adverse events. Further studies are required to examine objective clinical outcomes, based on advanced methodology, and explore the barriers against lifestyle modification in qualitative studies involving long-term follow-up.

## Abbreviations

ACS: Acute coronary syndrome
AMI: Acute myocardial infarction
HRQoL: Health-related quality of life
MACE: Major adverse cardiac events
MCS: Mental component summary
PCI: Percutaneous coronary intervention
PCS: Physical component summary
QoL: Quality of life (QoL)
